# Airborne Immunity: A Bacterial Volatile Primes Plant Defenses by Unlocking a Phytoalexin Biosynthetic Checkpoint

**DOI:** 10.1111/pce.70816

**Published:** 2026-08-10

**Authors:** Ivan A. Paponov, Chidananda Nagamangala Kanchiswamy, Simone Bossi, Francesco Caldo, Ambra S. Parmagnani, Mickael Malnoy, Massimo E. Maffei

**Affiliations:** ^1^ Department of Food Science Aarhus University Aarhus Denmark; ^2^ Department of Life Sciences and Systems Biology, Plant Physiology Unit University of Turin Turin Italy; ^3^ Research and Innovation Centre Edmund Mach Foundation San Michele all'Adige Italy

**Keywords:** calcium signaling, microbial volatiles, ROS signalling, transcriptomics

## Abstract

Microbial volatile organic compounds (mVOCs) enable plants to perceive microbial activity prior to physical contact, yet the contribution of individual bacterial volatiles to immune signalling and disease resistance remains incompletely understood. Here, headspace GC–MS analysis demonstrates that the hemibiotrophic pathogen *Pseudomonas syringae* pv. *tomato* DC3000 (*Pst* DC3000) emits a distinct volatile blend containing the bacterium‐specific compounds 2‐methylbutanoic acid (2‐MBA) and 3‐methylbutanoic acid (3‐MBA), with 2MBA as a dominant component. Exposure of *Arabidopsis thaliana* to the complete *Pst* DC3000 mVOC blend induced extensive transcriptional reprogramming activation of pattern‐recognition receptor‐associated genes, MAP kinase signalling components, WRKY transcription factors, camalexin biosynthetic genes and early defense responses. The latter included cytosolic Ca^2+^ ([Ca^2+^]_cyt_) elevation, K^+^ channel activation, hydrogen peroxide accumulation, and nitric oxide production, which culminated in camalexin accumulation in wild‐type shoots and roots. In contrast, these early signaling and physiological responses were strongly attenuated in the camalexin‐deficient *pad3* mutant. Phenotypic priming assays confirmed that mVOC pre‐exposure enhances resistance against subsequent *Pst* DC3000 infection largely through this PAD3‐dependent mechanism, while exogenous camalexin administration proved independently sufficient to restore robust pathogen protection, indicating that PAD3‐dependent camalexin biosynthesis contributes substantially, but not exclusively, to volatile‐induced resistance. Application of synthetic 2‐MBA, and to a lesser extent 3‐MBA, was sufficient to recreate rapid [Ca^2+^]_cyt_ elevation and ROS production. Quantitative expression profiling revealed that synthetic 2‐MBA modulates gene expression in an organ‐specific manner, upregulating shoot volatile perception and salicylic acid pathways, inducing root calcium and immune responses, and systemically activating auxin signaling and tryptophan biosynthetic genes. Together, these findings identify 2‐MBA as a primary active component of the *Pst* DC3000 volatile blend and indicate that full volatile‐induced defence depends on PAD3‐dependent camalexin accumulation together with integration of multiple volatile signals. Our results reveal how plants integrate distinct bacterial volatiles to trigger early signaling and coordinate localized and systemic camalexin‐dependent immunity.

## Summary statement

In *Arabidopsis thaliana*, bacterial volatiles from *Pseudomonas syringae* DC3000, dominated by 2‐methylbutanoic acid (2‐MBA), induce Ca^2+^, K+, ROS, and NO responses and transcriptional reprogramming to prime PAD3‐dependent camalexin accumulation and enhanced resistance.

## 1 Introduction

Plants, as sessile organisms, have evolved sophisticated immune systems to defend against a diverse array of microbial invaders. A cornerstone of plant immunity is the recognition of conserved microbial signatures, termed pathogen‐associated molecular patterns (PAMPs). This recognition is mediated by pattern recognition receptors (PRRs), typically located at the plant cell surface. These receptors, including receptor‐like kinases and receptor‐like proteins (RLPs), initiate pattern‐triggered immunity (PTI), a multilayered signalling network that restrict colonization (Ngou et al. 2024; Ngou et al. 2022; Nabi et al. 2024). PTI is characterized by rapid ion fluxes, particularly cytosolic Ca^2+^ ([Ca^2+^]_cyt_) elevation, production of reactive oxygen species (ROS), activation of mitogen‐activated protein kinase (MAPK) cascades, and extensive transcriptional reprogramming, ultimately leading to reinforcement of defence barriers and activation of antimicrobial responses (Millet et al. 2010; Yu et al. 2017; Mittler et al. 2022). Among the downstream outputs of PTI, phytoalexins play a crucial role in disease resistance, and disruption of their biosynthetic pathways markedly increases plant susceptibility to pathogens (Monsalvo et al. 2024).

While contact‐dependent immune signalling has been extensively studied, plants can also perceive microbial activity before physical interaction occurs. Microbial volatile organic compounds (mVOCs) act as airborne chemical cues that enable long‐distance communication between microbes and plants (Vlot and Rosenkranz 2022). These compounds are chemically diverse, encompassing alcohols, ketones, benzenoids, terpenoids, sulfur‐containing compounds, and alkenes (Netzker et al. 2020), and have been shown to influence plant growth, development, and defence‐related processes (Parmagnani et al. 2023). Accumulating evidence indicates that mVOCs can activate immune‐related pathways and prime resistance against subsequent pathogen challenge (Ryu et al. 2004; Bailly and Weisskopf 2012). Exposure to mVOCs has been reported to modulate plant hormone signalling networks, including salicylic acid, jasmonic acid, and auxin pathways, which integrate growth and defence regulation (Han et al. 2006; Cellini et al. 2018; Monti et al. 2023). Moreover, mVOCs can elicit classical PTI‐associated responses, such as [Ca^2+^]_cyt_ elevation, ROS production, and MAPK activation, indicating that airborne microbial cues can converge on canonical immune signalling pathways (Stael et al. 2015; Parmagnani et al. 2023).

Despite these advances, the functional significance of mVOC‐induced signalling remains incompletely resolved. Numerous studies have documented transcriptional, physiological, or developmental responses following mVOC exposure, yet whether these responses translate into effective disease resistance is often unclear. In particular, the mechanistic links between early mVOC‐induced signalling events and downstream defence execution remain poorly defined. It is unknown whether volatile‐triggered [Ca^2+^]_cyt_, ROS, and MAPK signalling reaches sufficient amplitude or duration to activate antimicrobial metabolism and establish a primed immune state. Clarifying how airborne microbial cues are converted into functional defence outputs is therefore essential for understanding the biological relevance of mVOC‐mediated plant–microbe interactions (Russo et al. 2022; Weralupitiya et al. 2024).

In *Arabidopsis thaliana*, the indole‐derived phytoalexin camalexin represents a major antimicrobial defence compound produced downstream of immune signalling. Camalexin biosynthesis integrates MAPK activity and WRKY transcriptional regulation and is required for resistance to a broad range of bacterial and fungal pathogens (Glawischnig et al. 2004; Ren et al. 2008; Li et al. 2022). Importantly, impairment of camalexin biosynthesis compromises defence even when upstream immune signalling remains partially active, highlighting the central role of secondary metabolism in defence execution (Bednarek 2012). Whether mVOC‐induced immune signalling requires camalexin biosynthesis to translate airborne cue perception into functional defence priming has not been systematically tested.

*Pseudomonas syringae* pv. *tomato* DC3000 (*Pst* DC3000) is a well‐established hemibiotrophic pathogen and a widely used model for dissecting plant immune responses. In addition to contact‐dependent virulence factors, bacteria like *Pseudomonas* species can emit volatile metabolites. Across diverse bacteria, branched‐chain amino acid catabolism is a well‐established source of methyl‐branched aldehydes and acids that may occur as volatile or semi‐volatile microbial products (Beck et al. 2002). Chemical identification of pathogen‐derived mVOCs provides a foundation for dissecting their biological activity and distinguishing specific immune cues from background microbial volatiles (Kanchiswamy et al. 2015). However, natural mVOC emissions consist of complex and dynamic blends whose composition depends on microbial species, growth phase, and environmental conditions (Netzker et al. 2020; Raio 2024). While testing individual compounds is essential for establishing causality, single volatiles may activate only subsets of the responses elicited by complete blends, making it critical to distinguish early signalling sufficiency from full defence activation.

In this study, we investigated whether mVOCs emitted by *Pst* DC3000 function as airborne immune cues in *Arabidopsis thaliana*. We tested the hypotheses that (i) *Pst* DC3000 mVOCs activate early immune‐associated signalling pathways characteristic of PTI, (ii) PAD3‐dependent camalexin biosynthesis is required to translate volatile‐induced signalling into functional defence priming, and (iii) the bacterium‐specific volatile 2‐methylbutanoic acid (2‐MBA) contributes to early immune signalling. To address these questions, we combined transcriptome analysis, genetically encoded biosensors, phytoalexin quantification, and infection assays to link volatile perception with immune signalling and disease resistance outcomes.

## 2 Materials and Methods

### 2.1 Plant Material and mVOC Co‐Cultivation System

*Arabidopsis thaliana* Columbia‐0 (Col‐0) and the Arabidopsis camalexin‐deficient *pad3* mutant (obtained from https://www.arabidopsis.org) were used in this study. Seeds were surface‐sterilized with 70% ethanol for 2 min followed by 5% calcium hypochlorite for 5 min and rinsed four times with sterile distilled water. Seeds were sown in a sealed headspace co‐cultivation system as previously described (Parmagnani et al. 2023). The upper compartment contained half‐strength Murashige and Skoog (MS) medium, while the lower compartment contained Nutrient Agar II (NA II; peptone from casein 3.5 g L^−1^, peptone from meat 2.5 g L^−1^, peptone from gelatin 2.5 g L^−1^, yeast extract 1.5 g L^−1^, NaCl 5 g L^−1^, agar 15 g L^−1^; pH 7.2).

Following a 3‐day vernalization at 4°C in darkness, seedlings were grown in a controlled environment chamber under a 16 h light/8 h dark photoperiod (≈70 μ molphotons m^−2^ s^−1^). *Pseudomonas syringae* pv. *tomato* DC3000 (*Pst* DC3000) (kindly provided by Dr. Ivan Baccelli, IPSP‐CNR, Florence, Italy) was introduced into the lower compartment, and seedlings were exposed to bacterial mVOCs for time periods ranging from 30 min to 24 h, depending on the downstream analysis. Roots and shoots were harvested separately, immediately frozen in liquid nitrogen, and stored at −80°C.

### 2.2 Transcriptome Analysis of Plants Exposed to *Pst* DC3000 mVOCs

Total RNA was extracted from frozen whole (roots and shoots) plants (100 mg) harvested after 16 h exposure to *Pst* DC3000 mVOCs. RNA isolation, quality control, and labeling were performed as previously described (Paponov et al. 2021), with RNA quantification further validated spectrophotometrically using a NanoDrop ND‐1000 (Thermo Fisher Scientific). Four independent biological replicates were used.

Double‐stranded cDNA was synthesized from 500 ng total RNA and transcribed into antisense cRNA, which was labeled with Cy3 or Cy5 dyes. Equal amounts (825 ng) of labeled control and treated cRNA were hybridized to Agilent *Arabidopsis thaliana* 4×44 K v3 microarrays using a two‐color design, following MIAME guidelines (Brazma et al. 2001). Microarrays were scanned and processed as described previously (Paponov et al. 2021). The complete dataset is deposited in the NCBI Gene Expression Omnibus under accession number GSE281883.

### 2.3 Quantitative RT–PCR Analysis

Gene expression was validated by quantitative RT–PCR using a QuantStudio 3 Real‐Time PCR System (Applied Biosystems). cDNA was synthesized from DNase‐treated RNA, and qPCR reactions were performed using Maxima SYBR Green/ROX Master Mix (Thermo Fisher Scientific) in a final volume of 25 μL.

Thermal cycling conditions consisted of 50°C for 2 min, 95°C for 10 min, followed by 40 cycles of 95°C for 15 s and 60°C for 60 s. Primer sequences are listed in Table S1. Among the tested reference genes (*ACT2*, *eEF1Bα2*, *UBP6*), *UBP6* was selected for normalization based on expression stability. Relative transcript levels were calculated using normalized Ct values.

### 2.4 Fluorescence‐Based Analysis of Early Immune Signalling

Early immune‐associated signalling responses were monitored in shoots and roots using fluorescence‐based probes as described previously (Maffei et al. 2006; Mithöfer et al. 2009). [Ca^2+^]_cyt_ dynamics were assessed using Calcium Orange (5 μM), following exposure to *Pst* DC3000 mVOCs for 30 min to 3 h. Fluorescence was detected using a Nikon Eclipse C1 confocal microscope with a Krypton/Argon laser with excitation at 488 nm and emission collected at 500–540 nm. Control samples consisted of unexposed tissues treated with 5 µM calcium orange solution.

Voltage‐ and ligand‐gated K^+^ channel activity was measured using the FluxOR™ Potassium Ion Channel Assay Kit (Invitrogen), following the manufacturer's protocol and previous reports (Bricchi et al. 2013).

Steady state hydrogen peroxide production was quantified using Amplex Red (10‐acetyl‐3,7‐dihydroxyphenoxazine), and scans were recorded using an Argon laser (458 nm/5 mW; 476 nm/5 mW; 488 nm/20 mW; 514 nm/20 mW) and HeNe lasers (543 nm/1.20 mW and 633 nm/10 mW). Fluorescence measurements were taken from 30 min to 24 h, as previously described (Maffei et al. 2006). Nitric oxide (NO) accumulation was detected using 4,5‐diaminofluorescein diacetate (DAF‐FM DA), an argon laser was used at an excitation wavelength of 488 nm, and the emitted light was captured through a band‐pass filter with a range of 508–525 nm. Fluorescence was recorded from 30 min to 24 h after *Pst* DC3000 mVOC exposure. Where indicated, tissues were pre‐treated with the NO scavenger cPTIO (1 mM) prior to staining.

### 2.5 In Vivo Imaging of Ca^2+^ and Glutathione Redox Dynamics in Response to 2‐MBA

Four‐week‐old *A. thaliana* plants expressing the genetically encoded biosensors R‐GECO1 (Keinath et al. 2015b) or Grx1‐roGFP2 (Meyer and Dick 2010) kindly provided by Prof. Alex Costa (NOLIMITS Unitech) were used to monitor Ca^2+^ and glutathione redox dynamics, respectively. Plants were grown in soil under controlled conditions (16/8 h photoperiod, 23°C).

Synthetic 2‐methylbutanoic acid (2‐MBA; Merck) and 3‐methylbutanoic acid (3‐MBA; Merck) were dissolved in DMSO and applied (1000 μg) to a 2 × 2 cm filter paper placed adjacent to the plants without direct contact. DMSO served as control. R‐GECO1 was excited at 580 nm using a CoolLED pE‐300 ultra. Grx1‐roGFP2 was excited sequentially at 405 nm (405/40 nm) and 488 nm (470/40 nm), and emissions were detected at 505–530 nm with a FITC/GFP filter. Images were captured every 10 s with a Nikon DS‐Fi3 Mono Camera (1 s exposure, 8 bit 1440 × 1024 dpi). For R‐GECO1, changes in fluorescence (Δ*F*/*F*_0_) were determined by normalizing the GFP emission (*F*) of selected ROIs to the initial fluorescence (*F*_0_) and plotting over time. For Grx1‐roGFP2, changes in the 405/488 nm emission ratio (Δ*R*/*R*_0_) were calculated by normalizing the ratio (*R*) of analyzed ROIs to the initial ratio (*R*_0_) and plotting over time. To correct background noise, fluorescence was measured in a region outside the sample and subsequently subtracted from each channel before calculating fluorescence or ratios.

### 2.6 Camalexin Quantification

Camalexin was extracted from frozen shoot and root tissue using 80% methanol and quantified by ultra‐performance liquid chromatography–high‐resolution mass spectrometry (UPLC‐HRMS) as described previously (Glawischnig et al. 2004). Identification was based on retention time and mass spectra comparison with a commercial camalexin standard (Sigma‐Aldrich). Quantification was performed using an external calibration curve.

### 2.7 Analysis of GUS Reporter Genes Expression

*A. thaliana* Col0 WRKY33:GUS lines were obtained from https://www.arabidopsis.org. To assess GUS reporter activity following exposure to *Pst* mVOCs, roots and leaves from both wild‐type and transgenic plants were subjected to a 2‐h incubation at 37°C in a GUS staining solution. The staining buffer comprised 10 mM EDTA, 0.1% Triton X‐100, 2 mM K_3_[Fe(CN)_6_], 2 mM K_4_[Fe(CN)_6_], and 1 mg/mL X‐Gluc, all within a 50 mM NaH_2_PO_4_ buffer at pH 7.0 (Dedow et al. 2022). Stained samples were observed and photographed with a microscope (Nikon).

### 2.8 Priming Assay and Bacterial Infection

Four‐week‐old Arabidopsis wild‐type (WT) and *pad3* plants were exposed to *Pst* DC3000 mVOCs for 24 h in sealed glass containers under controlled conditions. Following priming, plants were inoculated with *Pst* DC3000 (OD_600_ = 0.08; ~3 × 10^7^ CFU mL^−1^) by syringe infiltration of fully expanded leaves and maintained at high relative humidity (~90%). Disease symptoms were assessed at 3–4 days post‐inoculation (dpi). For bacterial titer determination, infiltrated leaf tissue was harvested at 3 dpi, weighed, and homogenised in 10 mM MgCl_2_ (10 mL per sample). Homogenates were serially diluted 1:10 in 10 mM MgCl_2_, and 20 µL of the dilution yielding a countable number of colonies (3–30 per spot) was plated on King's B agar supplemented with rifampicin (50 µg mL^−1^). Colonies were counted after 2 days at 28°C. Bacterial titer was expressed as CFU per mg fresh weight [CFU mg^−1^ = mean colony count × (dilution volume/plated volume) × 10^*n*^/leaf fresh weight, where *n* is the dilution step counted] and log_10_‐transformed prior to analysis. Titers were determined for *n* = 3 leaves per genotype and treatment in a single experiment.

### 2.9 Headspace GC–MS Analysis of *Pst* DC3000 mVOCs

Volatile compounds emitted by *Pst* DC3000 were collected using stir bar sorptive extraction and analysed by GC–MS following established protocols (Parmagnani et al. 2023). Volatiles were identified by comparison with NIST mass spectral libraries, Kovàts retention indices, and authentic standards.

### 2.10 Camalexin Chemical Complementation of *pad3* (Sufficiency Assay)

To determine whether exogenous camalexin is sufficient to restore *Pst* DC3000 resistance in the camalexin‐deficient *pad3* background, purified camalexin (analytical standard; Sigma‐Aldrich) was dissolved in DMSO (50 mM stock) and diluted in 10 mM MgCl_2_ to working concentrations of 1, 10, 25, and 50 µM (final DMSO ≤ 0.1% v/v in all solutions, including controls). Leaves of 4‐week‐old *pad3* plants were pressure‐infiltrated with camalexin, mock buffer (10 mM MgCl_2_), or the 0.1% DMSO solvent control using a needleless syringe. Twenty‐four hours later, leaves were challenged with *Pst* DC3000 at OD_600_ = 0.08, the inoculum density used throughout Figure 1, and bacterial titre was quantified at 3 dpi as described in section 2.8 (CFU mg^−1^ fresh weight, log_10_‐transformed). Titres were determined from three replicates of leaves per dose. A camalexin‐only control (50 µM, mock‐camalexin, no pathogen challenge) was included to assess direct phytotoxicity. This assay evaluated camalexin sufficiency in the absence of volatile priming; the combined camalexin‐plus‐mVOC treatment was not part of this dataset.

### 2.11 Statistical Analysis

Data are presented as means ± SD from at least three independent biological replicates. For image‐based disease‐severity analysis, statistical differences among treatments were analyzed using one‐way ANOVA followed by Tukey's HSD test, while genotype‐by‐priming interactions were evaluated via two‐way ANOVA (*α* = 0.05) using statsmodels and SciPy. Data are presented as mean ± SEM. Microarray data were analysed using GeneSpring and the R/Bioconductor package limma (Gentleman et al. 2006); differentially expressed genes (DEGs) were identified in GeneSpring, functional enrichment used Gene Ontology annotations from TAIR, and protein‐association networks were generated with STRING v12 (Szklarczyk et al. 2023). For the camalexin dose–response assay, each dose was compared with the untreated pad3 + Pst reference by Welch's *t*‐test, and the dose series (1–50 µM) was additionally tested for a linear trend against log‐transformed dose.

## 3 Results

### 3.1 Pre‐Exposure to *Pst* DC3000 mVOCs Alters Disease Symptom Development in a PAD3‐Dependent Manner

To determine whether bacterial volatiles affect disease outcome, Arabidopsis WT and *pad3* plants were exposed for 24 h to the *Pst* DC3000 mVOCs and subsequently infiltrated with *Pst* DC3000 (OD_600_ = 0.08). Disease symptoms were evaluated 3 days post‐inoculation. Representative leaves are shown in Figure 1. Figure 1A shows WT control healthy leaves. WT plants pre‐exposed to *Pst* DC3000 mVOCs showed a reduced chlorosis and tissue collapse (Figure 1C) compared with *Pst* DC3000‐exposed controls (Figure 1B). Leaves of *Pst* DC3000 mVOC‐pretreated WT plants and infected with *Pst* DC3000 (Figure 1D) displayed reduced necrotic areas with respect to conttrols infected by *Pst* DC3000 (Figure 1B). WT plant pre‐treated with 2‐MBA and then infected by *Pst* DC3000 (Figure 1E) showed a reduced necrosis when compared to *Pst* DC3000 infected control plants (Figure 1B). Disease severity shows a significant reduction upon priming with *Pst* mVOC and 2.MBA, with respect to controls (Figure 1K). The bacterial titre, measured in the same experiment shown in Figure 1A–J, confirmed that volatile priming reduces *Pst* DC3000 growth in wild‐type plants (Figure 1L). Relative to non‐primed, infected plants, mVOC priming lowered titre approximately 97‐fold, and priming with 2‐MBA alone lowered it approximately 10‐fold, consistent with 2‐MBA acting as a partial, biologically active component of the full blend (see below).

**FIGURE 1:**
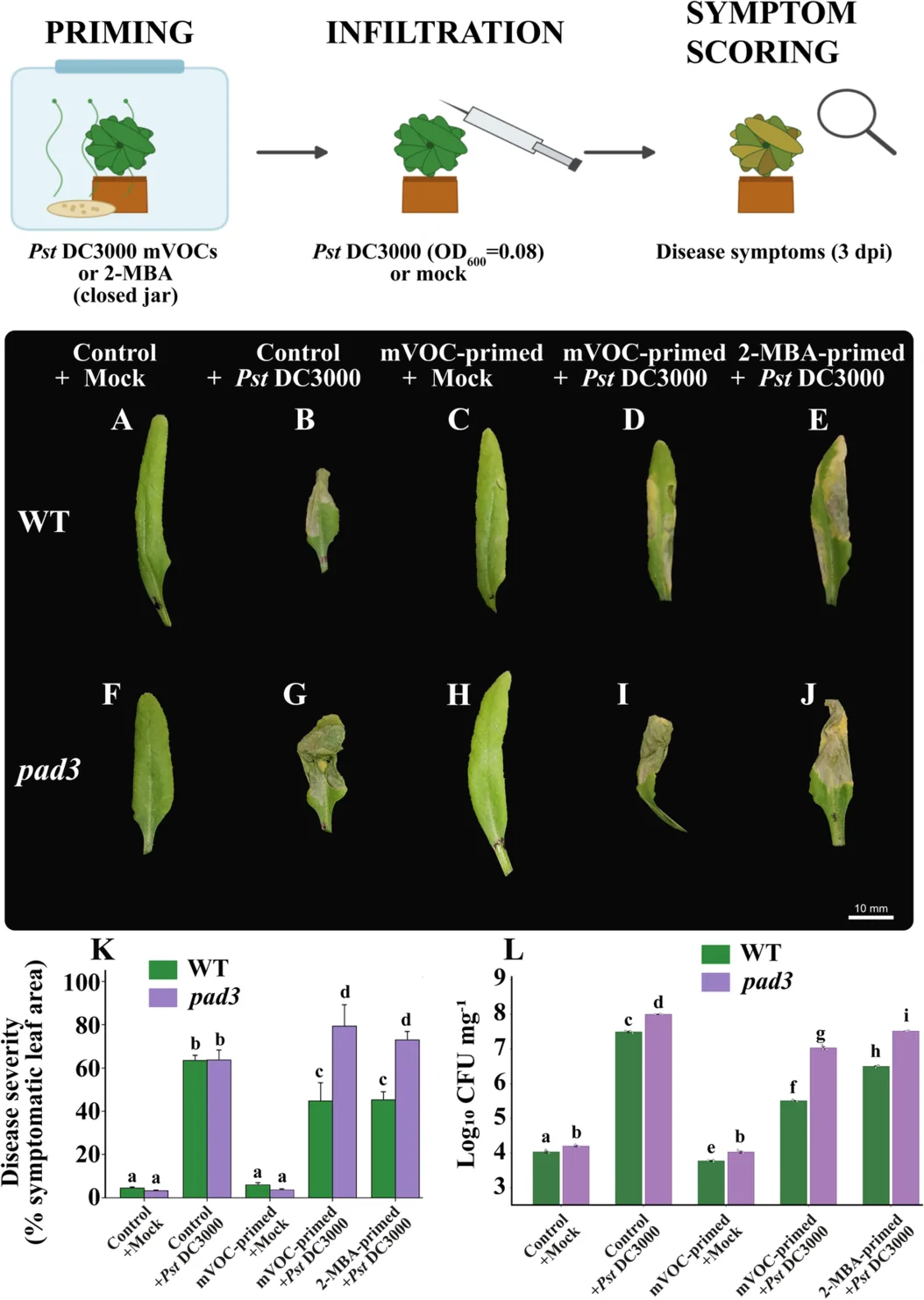
Experimental design and phenotypic response of *Arabidopsis thaliana* wild‐type (Col‐0) and *pad3* plants to volatile priming followed by *Pst* DC3000 infection, with quantitative image analysis of disease severity. (Top) Schematic of the experimental workflow. (A–J) Representative leaves from WT (A–E) and *pad3* (F–J) plants under five conditions. (K) Disease severity quantified from RGB leaf images as the percentage of segmented leaf area showing symptoms (chlorosis + necrosis/tissue collapse). The genotype × priming interaction was significant (two‐way ANOVA, *p* = 0.039), indicating that the protective effect of volatile priming on symptom development is partly PAD3‐dependent. (L) In planta bacterial growth at 3 dpi. Bars indicate standard deviation; different letters indicate significant differences (Tukey HSD, *p* < 0.05), scale bar = 10 mm.

In *pad3*, priming remained protective against infection but to a markedly lesser extent than in WT (Figure 1H). mVOC and 2‐MBA priming reduced the titre approximately 10‐fold and threefold, respectively, both substantially smaller than the corresponding WT effects (Figure 1L). This contrasts with the disease‐severity (Figure 1K), in which primed *pad3* leaves appeared more symptomatic than non‐primed, infected *pad3* leaves. Considered together with the severity data, the titre results indicate that PAD3‐dependent camalexin biosynthesis accounts for most, but not all, of the protection conferred by priming, and that CFU and visual severity scoring diverge specifically in *pad3*.

To determine whether camalexin alone, in the absence of volatile priming, is sufficient to confer *Pst* DC3000 resistance, synthetic camalexin was infiltrated into *pad3* leaves before pathogen challenge, using the same OD_600_ = 0.08 inoculum as in Figure 1. Every camalexin dose tested (1, 10, 25, and 50 µM) reduced bacterial titre relative to untreated *pad3* + *Pst* DC3000, by approximately three to four orders of magnitude across the dose range (Figure S1). The response was not monotonic between 1 and 50 µM: the lowest dose already produced close to the maximal reduction observed at higher doses. Camalexin is therefore sufficient, on its own, to restore *Pst* DC3000 resistance in the camalexin‐null background, supporting the camalexin‐dependent defence role.

### 3.2 *Pst* DC3000 mVOCs Induce Extensive Transcriptional Changes

To characterize the molecular response to *Pst* DC3000 volatiles, we performed a whole‐plant transcriptomic analysis of Arabidopsis plants harvested 16 h after exposure to the *Pst* DC3000 mVOC blend. A total of 43,663 transcripts were detected. The volcano plot in Figure 2A illustrates the distribution of transcriptional changes, with significantly up‐ or down‐regulated genes highlighted (using corrected *p* values). Applying a threshhold of |log_2_ fold change| ≥ 1 identified 182 DEGs (see Data Set S1).

**FIGURE 2:**
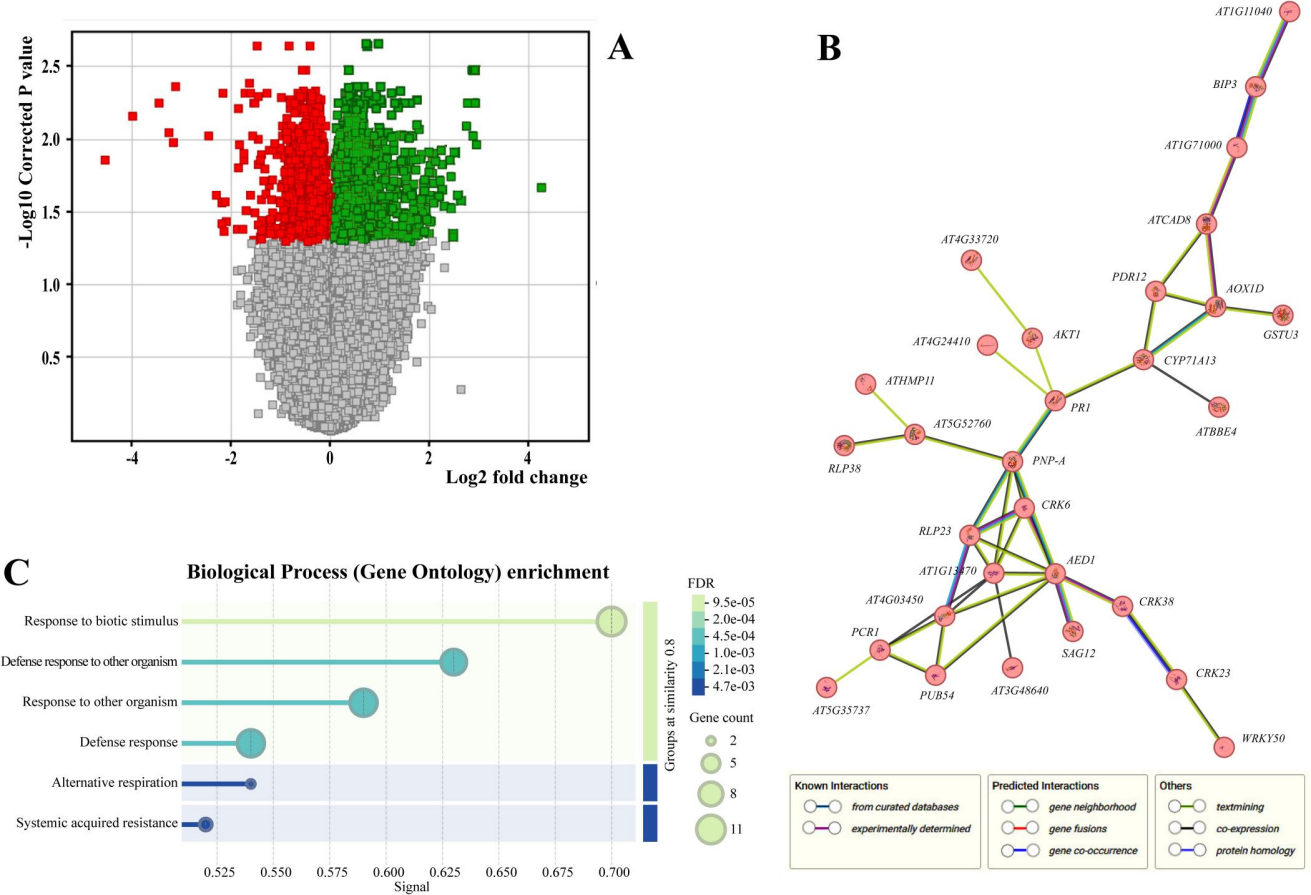
Effects of *Pst* DC3000 mVOCs on the Arabidopsis transcriptome and STRING network visualization of differentially expressed genes (DEGs). (A) Volcano plot from microarray‐based gene expression analysis showing up‐ and down‐regulated genes. (B) Protein association network of DEGs generated with STRING (Szklarczyk et al. 2023); nodes represent proteins encoded by DEGs and are clustered by k‐means; edges indicate functional associations (color‐coded as shown in the legend). (C) Gene Ontology (GO) enrichment analysis of biological processes and their statistical significance. [Color figure can be viewed at wileyonlinelibrary.com]

Protein‐protein interaction analysis using STRING on this filtered gene set revealed interconnected clusters (Figure 2B), including receptor‐like proteins (RLP23, RLP38), cysteine‐rich receptor‐like kinases (CRK6, CRK23, CRK38), defence‐related proteins (PR1, AED1, SAG12), and transcriptional factors such as WRKY50, redox‐related enzymes (GSTU3, AOX1D), transporters, including PDR12, and camalexin‐associated genes (such as CYP71A13). Gene Ontology enrichment analysis (Figure 2C) showed significant over‐representation of biological processes related to response to biotic stimulus, defence response, and response to chemical stimulus (Table S2 lists the genes clustered by STRING, together with hub‐gene connection node numbers; Supporting Data Set S1 provides the full DEG list with associated GO terms).

### 3.3 *Pst* DC3000 mVOCs Activate the WRKY33–Camalexin Pathway, Leading to Camalexin Accumulation in Wild Type but Not in *pad3*

Camalexin levels were quantified by UPLC‐HRMS after 16 h of *Pst* DC3000 mVOC exposure (Figure 3A). Camalexin accumulated in both shoots and roots of WT plants, whereas none was detectable in *pad3*. Time‐course RT‐qPCR analysis revealed induction of the camalexin biosynthetic genes *CYP79B2*, *CYP79B3*, *CYP71A13*, and *PAD3* in response to *Pst* DC3000 mVOC exposure (Figure 3B–E). Upstream regulatory components *MPK3*, *MPK6*, and *WRKY33* were also induced in both genotypes, albeit with genotype‐specific temporal patterns (Figure 3F–H). Analysis of WRKY33:GUS reporter lines showed expanded GUS staining in cotyledons after *Pst* DC3000 mVOC exposure, extending from major veins into the surrounding mesophyll (Figure 3I,J), whereas root staining patterns remained largely unchanged (Figure 3K,L).

**FIGURE 3:**
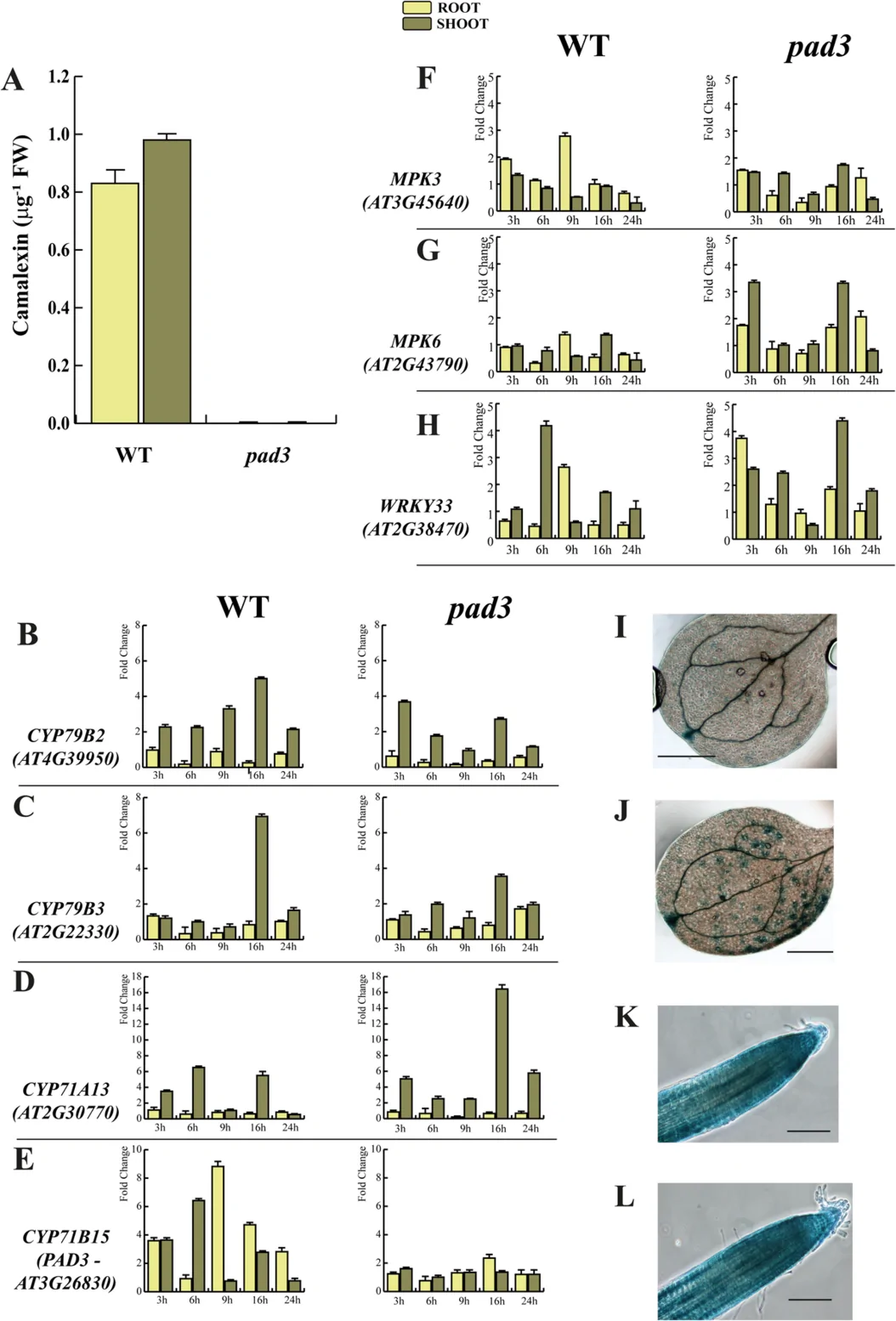
*Pst* DC3000 mVOCs activate camalexin biosynthesis and WRKY33 expression in Arabidopsis WT and genes involved in plant response to camalexin biosynthesis in WT and *pad3*. (A) Camalexin quantification in shoots and roots of WT and *pad3* plants. (B–E) Time‐course expression of camalexin biosynthetic genes in roots and shoots. (F–H) Expression of upstream regulatory genes *MPK3*, *MPK6*, and *WRKY33*. (I–L) WRKY33:GUS reporter activity in cotyledons (I, J) and roots (K, L) of control (I, K) versus *Pst* DC3000 mVOC‐exposed (J, L) WT plants. Scale bars: (I, J) 1.5 mm; (K, L) 100 µm. Error bars indicate standard deviation. [Color figure can be viewed at wileyonlinelibrary.com]

### 3.4 Early Ca^2+^/K^+^ and ROS/NO Signalling Triggered Responses to *Pst* DC3000 Mvocs Are Attenuated in *pad3*

K^+^ efflux and membrane‐potential changes are well‐documented early events closely coupled to Ca^2+^ influx and ROS production during PTI (Dreyer and Uozumi 2011; Kanchiswamy et al. 2026; Melotto et al. 2024). To examine early signalling events, [Ca^2+^]_cyt_ concentration, ligand‐gated and voltage‐gated K^+^ channel activity, H_2_O_2_ accumulation, and nitric oxide (NO) production were monitored in shoots and roots following *Pst* DC3000 mVOC exposure (Figure 4). In WT seedlings, exposure triggered time‐dependent increases in these parameters relative to untreated controls: voltage‐gated K^+^ channel activity increased at early time points, followed by ligand‐gated K^+^ channel activity, sustained [Ca^2+^]_cyt_ elevation, and progressive steady state H_2_O_2_ and NO accumulation (Figure 4). In *pad3* seedlings, the magnitude of these responses was markedly reduced: [Ca^2+^]_cyt_ elevation was strongly attenuated, activation of both K^+^ channel types was weaker, and steady state H_2_O_2_ and NO accumulation remained consistently lower than in WT across all time points (Figure 4).

**FIGURE 4:**
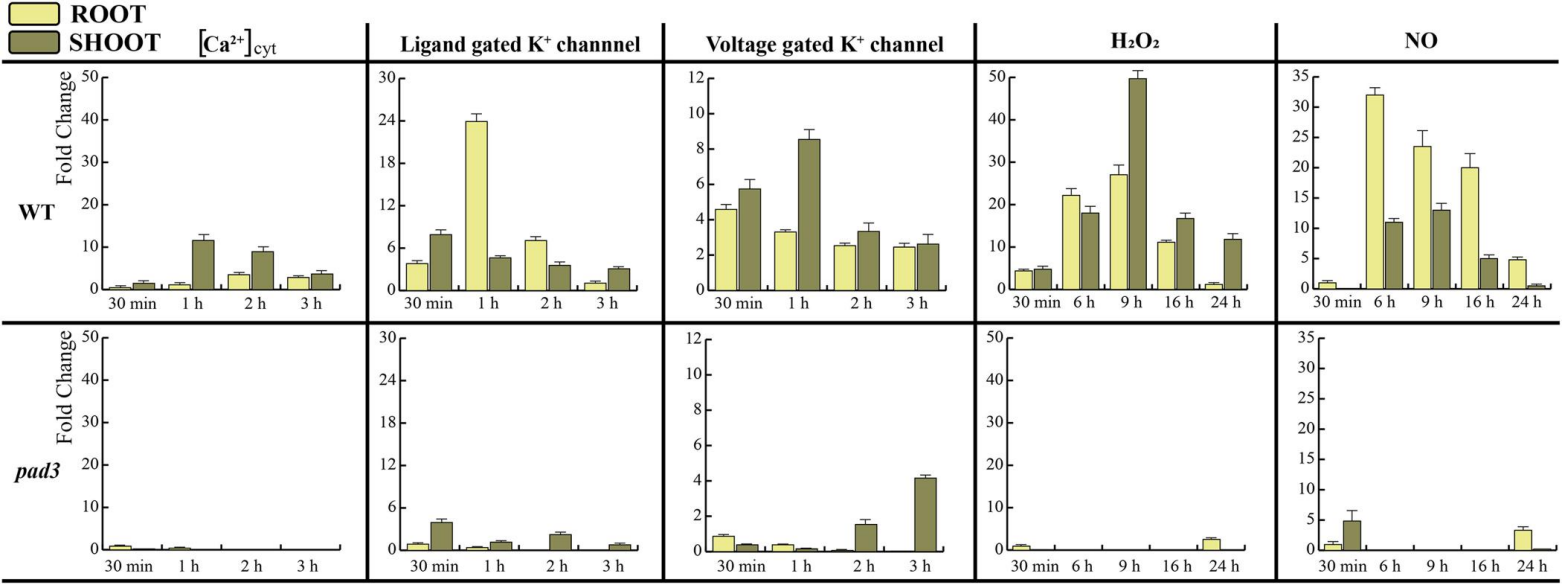
Early signaling responses to *Pst* DC3000 mVOCs in shoots and roots of *Arabidopsis thaliana* Col0 (WT) and *pad3* mutant seedlings. Data show fold changes in fluorescence intensity of cytosolic calcium, potassium channel activity, steady state H_2_O_2_ and NO production in *Pst* DC3000 mVOCs‐treated plants relative to untreated controls. Bars indicate standard deviation (*n* = 5). [Color figure can be viewed at wileyonlinelibrary.com]

### 3.5 GC–MS Identifies 2‐Methylbutanoic Acid and 3‐Methylbutanoic Acid as *Pst* DC3000‐Specific mVOCs

Headspace GC–MS analysis detected two confidently identified volatile compounds present in *Pst* DC3000 cultures but absent from sterile LB medium controls: 2‐methylbutanoic acid (2‐MBA) and 3‐methylbutanoic acid (3‐MBA) (Figure 5). Their identities were confirmed by comparison with authentic standards based on retention times and mass‐spectral fragmentation patterns and by matching against the NIST mass spectral library. Additional *Pst* DC3000‐specific peaks were detected but were not confidently identified and are regarded as candidates for future characterization. Because chromatographic peak abundance alone does not establish biological activity, both confidently identified *Pst* DC3000‐specific compounds were included in subsequent functional assays. 2‐MBA is emphasized below because it showed stronger biological activity than the structurally related isomer 3‐MBA.

**FIGURE 5:**
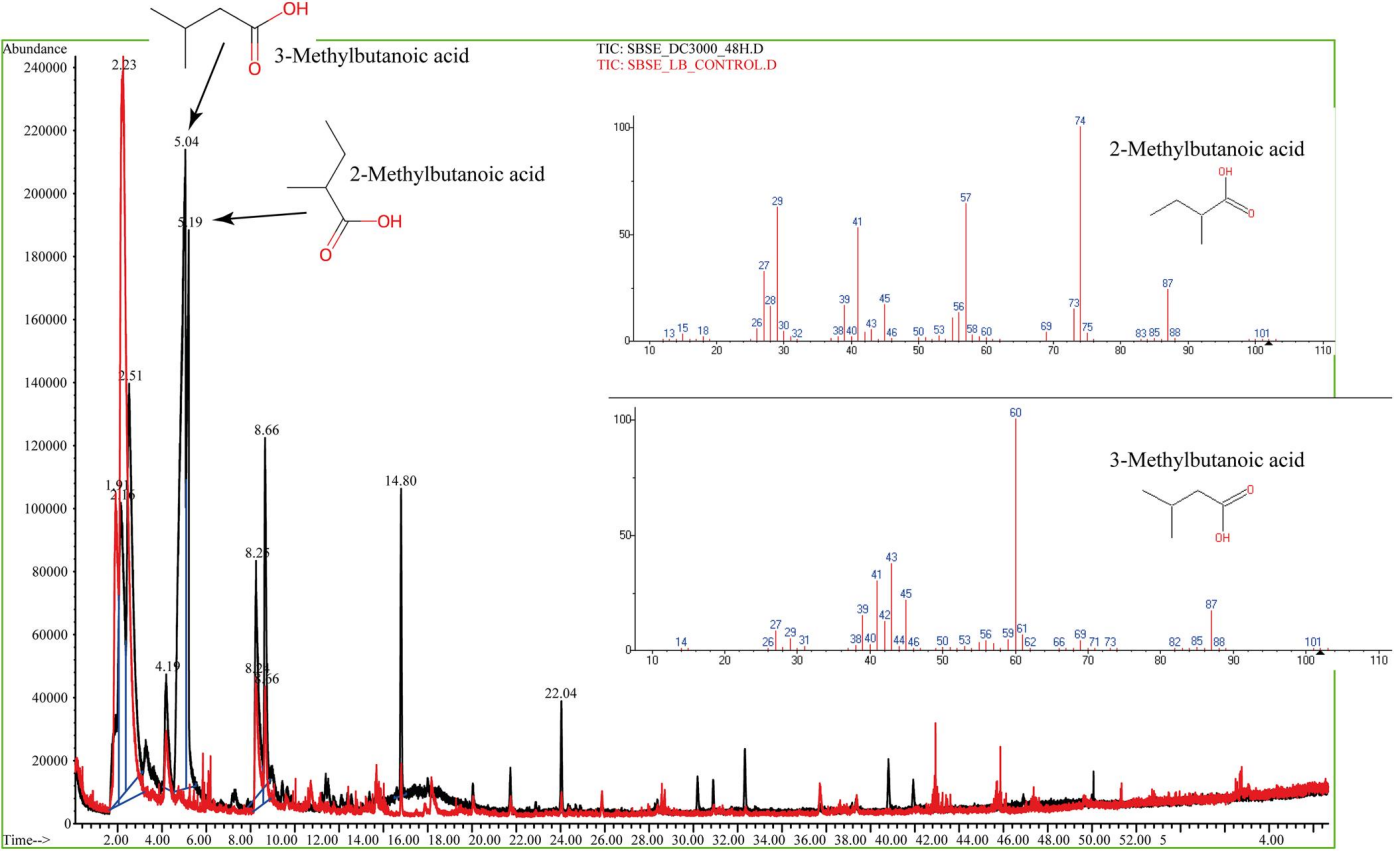
Head‐space GC‐MS total ion current chromatogram from *Pst* DC3000 (black line) and sterile LB medium control (red line). Peaks assigned to 2‐methylbutanoic acid (2‐MBA) and 3‐methylbutanoic acid (3‐MBA) are indicated. Inset shows the corresponding mass spectra and chemical structures of the two major compounds. Several peaks found in *Pst* DC3000 mVOCs that overlap with the medium volatiles were considered medium‐associated, whereas additional *Pst* DC3000‐specific minor peaks that could not be confidently assigned are treated as candidates for future characterization. [Color figure can be viewed at wileyonlinelibrary.com]

### 3.6 Synthetic 2‐MBA and 3‐MBA Induce [Ca^2+^]_Cyt_ Elevation Followed by Glutathione Redox Changes in Arabidopsis Leaves

To test whether 2‐MBA or 3‐MBA alone can trigger early responses, [Ca^2+^]_cyt_ and glutathione redox dynamics were monitored in vivo using the R‐GECO1 and Grx1‐roGFP2 biosensors, respectively (Figure 6). The intensiometric biosensor R‐GECO1, which is highly sensitive to changes of [Ca^2+^]_cyt_, allows live imaging and quantitative dynamic measurements (Keinath et al. 2015a). Time‐lapse imaging showed that application of synthetic 2‐MBA caused a rapid increase in R‐GECO1 fluorescence (Figure 6A), compared with DMSO controls (Figure 6C). 3‐MBA was also able to trigger changes of [Ca^2+^]_cyt_; however, the quantification of the maximal response (Δ*F*_max_/F_0_) confirmed significantly higher [Ca^2+^]_cyt_ elevations in 2‐MBA‐treated plants compared with both control and 3‐MBA‐trated plants (Figure 6D).

**FIGURE 6:**
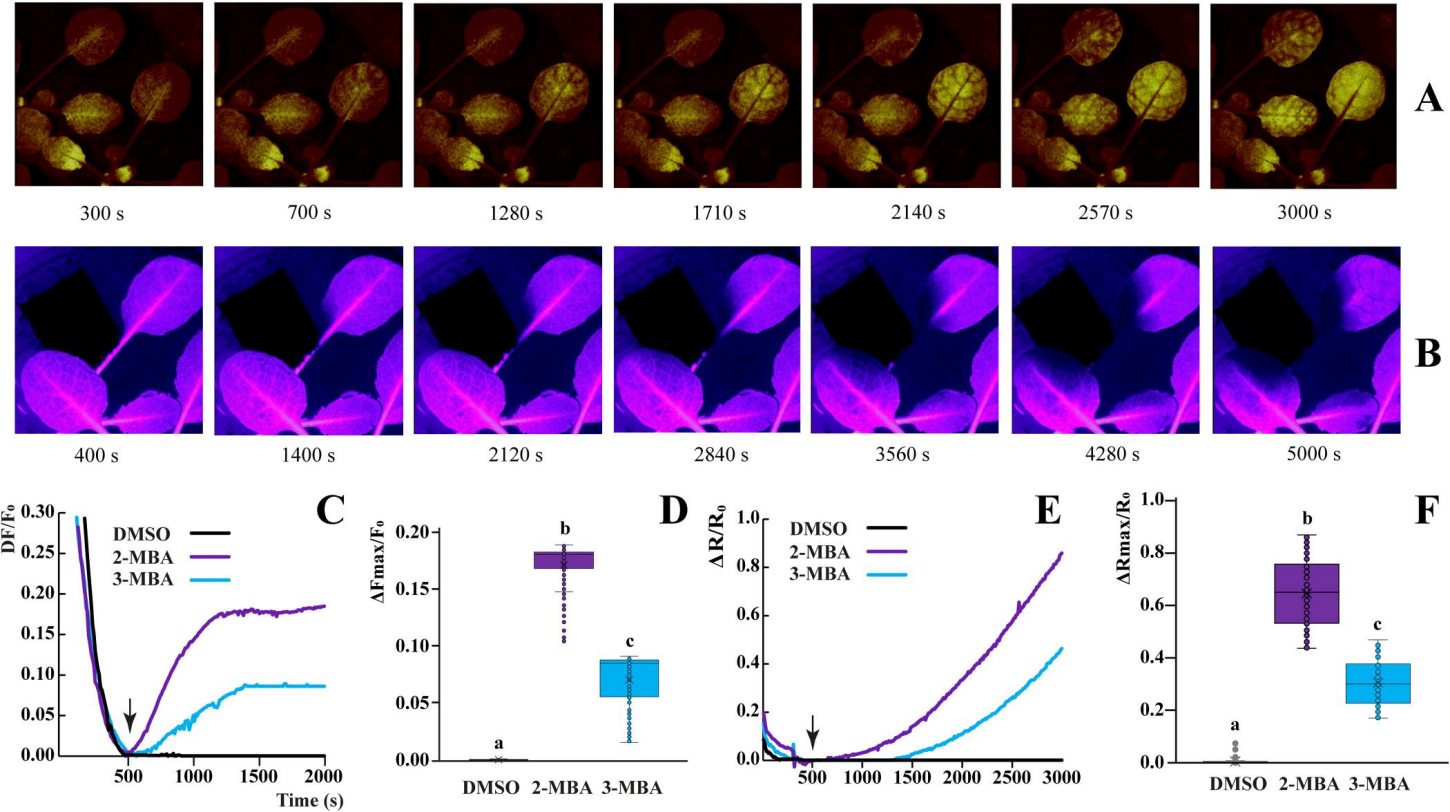
In vivo [Ca^2+^]_cyt_ and glutathione redox dynamics of *Arabidopsis thaliana* plants expressing R‐GECO1 and Grx1‐roGFP2 biosensors upon exposure to synthetic 2‐methylbutanoic acid (2‐MBA) and 3‐methylbutanoic acid (3‐MBA). Representative time‐lapse images of R‐GECO1 (A) and Grx1‐roGFP2 (B) fluorescence upon exposure to 2‐MBA. (C) Average normalized Δ*F*/*F*_0_ traces for [Ca^2+^]_cyt_. (D) Maximum [Ca^2+^]_cyt_ response (Δ*F*_max_/*F*_0_). (E) Average normalized 405/488 ratio traces for glutathione redox state. (F) Maximum redox change (Δ*R*_max_/*R*_0_). Error bars = SD; different letters indicate significant (*p* < 0.05) differences; arrows indicate the timing of 2‐MBA or 3‐MBA exposure. [Color figure can be viewed at wileyonlinelibrary.com]

Because Ca^2+^ waves are often accompanied by changes in cellular redox status, parallel experiments were performed using the Grx1‐roGFP2 sensor. Exposure to 2‐MBA increased the GSSG:GSH ratio in leaf tissue surrounding the application site (Figure 6B), indicating a shift toward a more oxidized glutathione redox state (Figure 6E). 3‐MBA also elicited redox changes, but quantitative analysis showed that 2‐MBA induced a significantly stronger response (Figure 6F).

These results demonstrate that both 2‐MBA and 3‐MBA elicit the early Ca^2+^ and redox signaling responses, with stronger effects observed for 2‐MBA.

### 3.7 Synthetic 2‐MBA Modulates Signalling and Biosynthetic Gene Expression in Arabidopsis

Following the assessment of the phenotypic effects of 2‐MBA, we investigated its impact on the molecular level by analyzing gene expression. Total RNA was extracted from the roots and shoots of *A. thaliana* (Col‐0) plants exposed for 24 h to either a DMSO solvent control or 100 ppm synthetic 2‐MBA dissolved in DMSO. Gene expression levels were quantified via qPCR and expressed as fold‐change values relative to the reference gene *UBP6*. To validate candidates from our transcriptomic analysis and target known physiological responses, we assayed a selection of marker genes involved in volatile organic compound (VOC) perception, hormone signaling, stress defense, and secondary metabolism.

We first evaluated a panel of genes associated with volatile perception and downstream hormonal signaling pathways. This was represented by genes implicated in (‐)‐germacrene D perception including *KARRIKIN INSENSITIVE 2* (*KAI2*), *MORE AXILLARY GROWTH 1* (*MAX1*), *MAX2*, *SUPPRESSOR OF MAX2 1* (*SMAX1*), the transcriptional co‐repressor TOPLESS (*TPL*) and *WRKY41*, together with components of ethylene signaling (*EIN2, ETR1*, *CTR1* and *WRKY22*), salicylic acid (SA) signaling (*SABP3* and *RLP23*) and brassinosteroid signaling (*BAK1*, *BRL1*, *BRL2*), and PAMP receptors (*FLS2*, *BIK1*, *XLG2*, *PUB26* and *WRKY29*). In roots, 2‐MBA exposure did not significantly alter the transcription of these targets relative to DMSO controls (Figure 7A). In contrast, treated shoots exhibited a significant upregulation of the volatile‐ and development‐related genes *KAI2*, *SMAX1*, and *MAX2*, along with the SA‐signaling component *SABP3* (Figure 7B).

**FIGURE 7:**
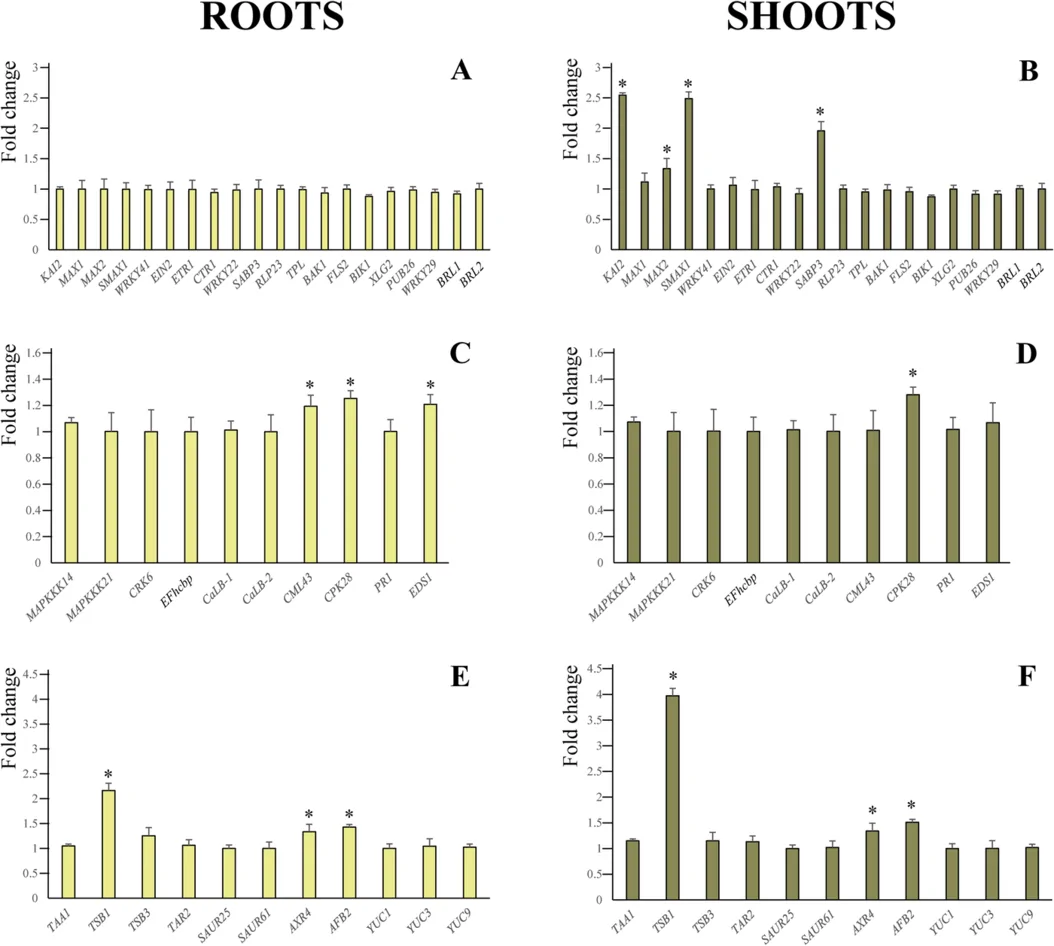
Transcriptional effects of DMSO and 2‐MBA exposure on *Arabidopsis thaliana*. Plants were treated with 2‐MBA dissolved in DMSO. DMSO‐treated plants served as controls. The effects of the treatments on gene expression were assessed for the following categories: 2‐MBA effect on VOC receptor genes in roots (A) and shoots (B). 2‐MBA effect on plant signaling, calcium, and defense‐related genes in roots (C) and shoots (D). 2‐MBA effect on genes involved in tryptophan biosynthesis and auxin‐related protein in roots (E) and shoots (F). Gene expression changes are presented as Log_2_ fold change normalized to the *UBP6* reference gene. Error bars indicate the standard deviation, asterisk indicates significant (*p* < 0.05) differences between 2‐MBA and DMSO treatments. [Color figure can be viewed at wileyonlinelibrary.com]

Next, we monitored transcript shifts in signal transduction cascades, calcium sensor dynamics, and core defense responses. This group encompassed MAPK kinase kinases (*MAPKKK14* and *MAPKKK21*), a cysteine‐rich receptor‐like kinase 6 (*CRK6*), calcium‐binding proteins (*EFhcbp*, *CaLB‐1*, *CaLB‐2*, *CML43*, and *CPK28*), and defense markers (*PR1*, *EDS1*, and *AED1*). In roots, 2‐MBA treatment significantly induced the expression of the calcium‐signaling genes *CML43* and *CPK28*, as well as the immune regulator *EDS1* (Figure 7C). In shoots, however, only *CPK28* showed a statistically significant increase in transcript abundance relative to the control (Figure 7D).

The final panel comprised loci governing tryptophan biosynthesis (*TAA1*, *TSB1*, *TSB3* and *TAR2*) and auxin signaling (*SAUR25*, *SAUR61*, *AXR4*, *AFB2*, *YUC1*, *YUC3* and *YUC9*). These targets provide a mechanistic link between the molecular data and the physiological observations, as tryptophan is the direct metabolic precursor of the phytoalexin camalexin, while auxin signaling governs root architecture and growth. Following 2‐MBA exposure, *TSB1* expression was upregulated in both organs, showing a distinctly robust induction in shoots (Figure 7E,F). Furthermore, the auxin response regulators *AXR4* and *AFB2* displayed moderate but significant transcript accumulation in both tissues (Figure 7E,F).

## 4 Discussion

Plants, as sessile organisms, rely on early detection of environmental cues to anticipate pathogen attack and adjust their defence state prior to physical contact. mVOCs represent an important class of airborne signals that enable plants to perceive microbial activity at a distance. Although numerous studies have demonstrated that mVOCs can influence plant growth, development, and defence‐related processes (Schulz‐Bohm et al. 2017; Weisskopf et al. 2021), the mechanisms by which volatile perception is translated into effective immune priming and disease resistance remain incompletely understood. Here, we show that mVOCs emitted by *Pseudomonas syringae* pv. *tomato* DC3000 are sufficient to activate canonical immune‐associated signalling, induce PAD3‐dependent camalexin accumulation, and prime resistance to subsequent pathogen challenge in *Arabidopsis thaliana*. Importantly, our data reveal a mechanistic separation between early volatile‐induced signalling and downstream defence execution, with camalexin biosynthesis acting as one of the critical metabolic checkpoints linking airborne cue perception to functional immunity.

### 4.1 *Pst* DC3000 mVOCs Activate Canonical Early Immune‐associated Signalling Pathways

A central finding of this study is that exposure to the *Pst* DC3000 mVOC blend rapidly activates early immune‐associated signalling events, including [Ca^2+^]_cyt_ elevation, altered K^+^ channel activity, and the production of ROS and nitric oxide (NO). These responses are hallmarks of early plant immune activation and are typically observed downstream of PRR engagement during MAMP perception (Defalco and Zipfel 2021; Köster et al. 2022). The coordinated activation of Ca^2+^, ROS, and NO observed here is consistent with their established roles as interconnected signalling modules that amplify immune outputs and shape downstream responses, while changes in K^+^ channel activity likely contribute by modulating membrane potential and Ca^2+^ signaling dynamics (Arasimowicz‐Jelonek and Floryszak‐Wieczorek 2016; Dreyer and Uozumi 2011).

Notably, these early signalling responses were reduced in the camalexin‐deficient *pad3* mutant. We interpret this result cautiously as evidence that the *pad3* background is associated with altered early responses to *Pst* DC3000 mVOCs, rather than as proof that camalexin biosynthesis directly controls upstream Ca^2+^, ROS, or NO signalling. One possible explanation is that PAD3‐dependent indolic metabolism contributes to the physiological state required for full signalling amplitude, consistent with reports that indole‐derived compounds, can influence ROS dynamics, cellular redox homeostasis, and the robustness of defence signalling (Yoshioka et al. 2009; Li et al. 2022). Alternatively, *pad3* may exhibit broader altered responses to volatile exposure or stress. Distinguishing between these possibilities will require targeted experiments, such as monitoring early Ca^2+^/ROS/NO responses after exogenous camalexin application. Our recent findings in the same pathosystem further support the importance of early ionic and redox modules in shaping long‐term plant responses, including changes in root system architecture (Kanchiswamy et al. 2026).

### 4.2 PAD3‐Dependent Camalexin Biosynthesis Contributes to Volatile‐Induced Immune Priming

Camalexin is a well‐characterised antimicrobial phytoalexin produced upon pathogen recognition, and activation of its biosynthetic machinery is generally considered a terminal output of immune signalling cascades (Glawischnig et al. 2004; Ren et al. 2008). Our results support an important contribution of PAD3‐dependent camalexin accumulation to mVOC‐mediated defence priming. Although immune‐associated transcriptional regulators, including MAP kinase‐associated signalling molecules and WRKY33, were activated following mVOC exposure, only wild‐type plants accumulated camalexin and showed the strongest reduction in subsequent *Pst* DC3000 growth. In *pad3*, volatile priming still reduced bacterial titre, but the effect was markedly weaker than in wild type, indicating that PAD3‐dependent camalexin biosynthesis accounts for a major part, but not all, of the protection conferred by priming. Infiltrating synthetic camalexin directly into *pad3* leaves prior to pathogen challenge fully restored robust resistance, reducing bacterial titre by three to four orders of magnitude across all tested doses, in agreement with literature data (Nguyen et al. 2022). This unambiguous rescue proves that the compromised defence phenotype in *pad3* is specifically driven by the lack of the camalexin metabolite itself, confirming its role as a functional executioner of volatile‐primed immunity. Our results point to a highly integrated, cooperative feedback loop where downstream metabolic flux coordinates with upstream signalling competence (Ca^2+^, K^+^, ROS, and NO)(Kanchiswamy et al. 2026). The metabolic synthesis of camalexin draws heavily on tryptophan precursors and heavily influences cellular redox balance, potentially via glutathione dynamics or metabolic shifting (Monsalvo et al. 2024). Studies are under way to determine whether camalexin is directly involved in the modulation of these early responses. In the *pad3* background, the disruption of this metabolic sink may create a negative feedback that suppresses early ion channel activation and oxidative bursts.

Therefore, this metabolic uncoupling indicates that transcriptional activation alone is insufficient to confer resistance and that metabolic commitment to camalexin biosynthesis represents an important checkpoint converting volatile perception into effective defence. Such a requirement for secondary metabolism to stabilise or reinforce immune outputs provides a mechanistic explanation for why volatile‐induced immune activation does not automatically translate into disease resistance. Similar principles have been proposed for defence priming triggered by non‐volatile cues, where the accumulation of specific defence metabolites is required to lock the system into a resistant state (Bednarek 2012; He et al. 2022).

### 4.3 2‐MBA Acts as a Biologically Active mVOC Signal and Induces Early Plant Responses

Chemical profiling identified 2‐MBA and 3‐MBA as *Pst* DC3000‐specific volatile compounds. This is consistent with previous evidence that methyl‐branched acids such as 2‐MBA and 3‐MBA can arise from bacterial branched‐chain amino acid catabolism (Beck et al. 2002). Functional comparison of the two structurally related isomers showed that both compounds can trigger Ca^2+^ elevation and redox responses, but 2‐MBA elicited significantly stronger responses. Thus, 2‐MBA should be considered a biologically active component of the *Pst* DC3000 mVOC blend.

The stronger response to 2‐MBA compared with 3‐MBA suggests that plant responses to methyl‐branched bacterial volatiles may depend on subtle structural differences between closely related compounds. However, the full immune effect of the natural *Pst* DC3000 volatile blend is unlikely to be explained by a single molecule alone. In natural environments, plants encounter complex mixtures of microbial volatiles whose composition varies with microbial identity, growth phase, and nutrient status (Kanchiswamy et al. 2015; Raio 2024). Combinatorial or synergistic interactions among volatile components may be required to stabilise early signalling outputs and promote sustained transcriptional and metabolic commitment to defence, consistent with previous observations that individual VOCs often elicit more restricted responses than complete volatile blends (Weisskopf et al. 2021).

Despite its relatively low volatility (vapor pressure of 0.067 kPa), 2‐MBA induced early plant responses, suggesting potential crosstalk between microbial volatile perception and pathways involved in environmental cue integration (Stirling et al. 2024). In addition, 2‐MBA induced transcriptional reprogramming, including upregulation of *KAI2*, *MAX2*, and *SMAX1* in shoot tissues. This expression profile is consistent with the role for KAI2/MAX2/SMAX1 module in perceiving airborne environmental signals (Varshney and Gutjahr 2023) and points to potential crosstalk with strigolactone‐related signaling pathway (Bythell‐Douglas et al. 2017). Such interactions might feed into broader environmental cue integration mechanisms involved in the processing of ambient VOCs as recently characterized (Stirling et al. 2024). Furthermore, the significant induction of the salicylic acid‐binding component *SABP3* in shoots, alongside calcium‐dependent transcripts (*CML43*, *CPK28*) and the defense regulator *EDS1* in roots, indicates that 2‐MBA perception triggers a multifaceted systemic response. The intersection of *Pst* mVOCs with these perception systems suggests a coordinated network in which volatile detection, calcium‐mediated transduction, and hormone signaling converge. This molecular cascade might contribute to downstream physiological and developmental responses, including the alterations observed in root architecture and primary root elongation. Ongoing investigations using comprehensive transcriptomic and metabolic profiling of both wild‐type and signaling‐deficient mutant lines will further elucidate the genetic architecture governing 2‐MBA‐responsive networks.

## 5 Conclusions and Outlook

Together, our findings support a model in which *Pst* DC3000 mVOCs function as airborne cues that initiate early signalling events in Arabidopsis, including Ca^2+^, redox, and transcriptional responses. Full defence activation and resistance to subsequent pathogen challenge depend on PAD3‐mediated camalexin biosynthesis and exposure to the complete volatile blend. 2‐MBA, a biologically active compounds emitted by *Pst* DC3000, triggers early ionic and redox signalling and may contribute to the immune‐associated transcriptional activation and defence responses induced by the complete mVOC blend. These findings reveal a functional separation between early signal perception and downstream metabolic commitment, underscoring the importance of secondary metabolism in translating volatile cues into effective defence.

This work advances our understanding of how plants interpret microbial volatiles and highlights the role of metabolic checkpoints in shaping defence outcomes. While experiments with synthetic 2‐MBA were essential to establish causal links between a defined bacterial volatile and early signalling responses, the full immune effects of *Pst* DC3000 mVOCs likely arise from the combined action of multiple volatile components. Elucidating how plants integrate complex volatile signatures and how these signals converge on metabolic decision points such as camalexin biosynthesis represents an important direction for future research, with potential implications for crop protection and disease management.

## Conflicts of Interest

The authors declare no conflicts of interest.

## Supporting information


Supporting File 1

Supporting File 2

Supporting File 3

Supporting File 3

## Data Availability

The data that support the findings of this study are available from the corresponding author upon reasonable request. Data are provided in Supporting data set and are available upon reasonable request.
