# Supplementary material for: Airborne Immunity: A Bacterial Volatile Primes Plant Defenses by Unlocking a Phytoalexin Biosynthetic Checkpoint
Source: Plant Cell Environ. 2026 Aug 10;49(11):9070–84. doi: 10.1111/pce.70816 (PMC13646668; doi:10.1111/pce.70816)
Supplement: Supplementary file 1 [file PCE-49-9070-s003.pdf]

Supplementary Fig. S1

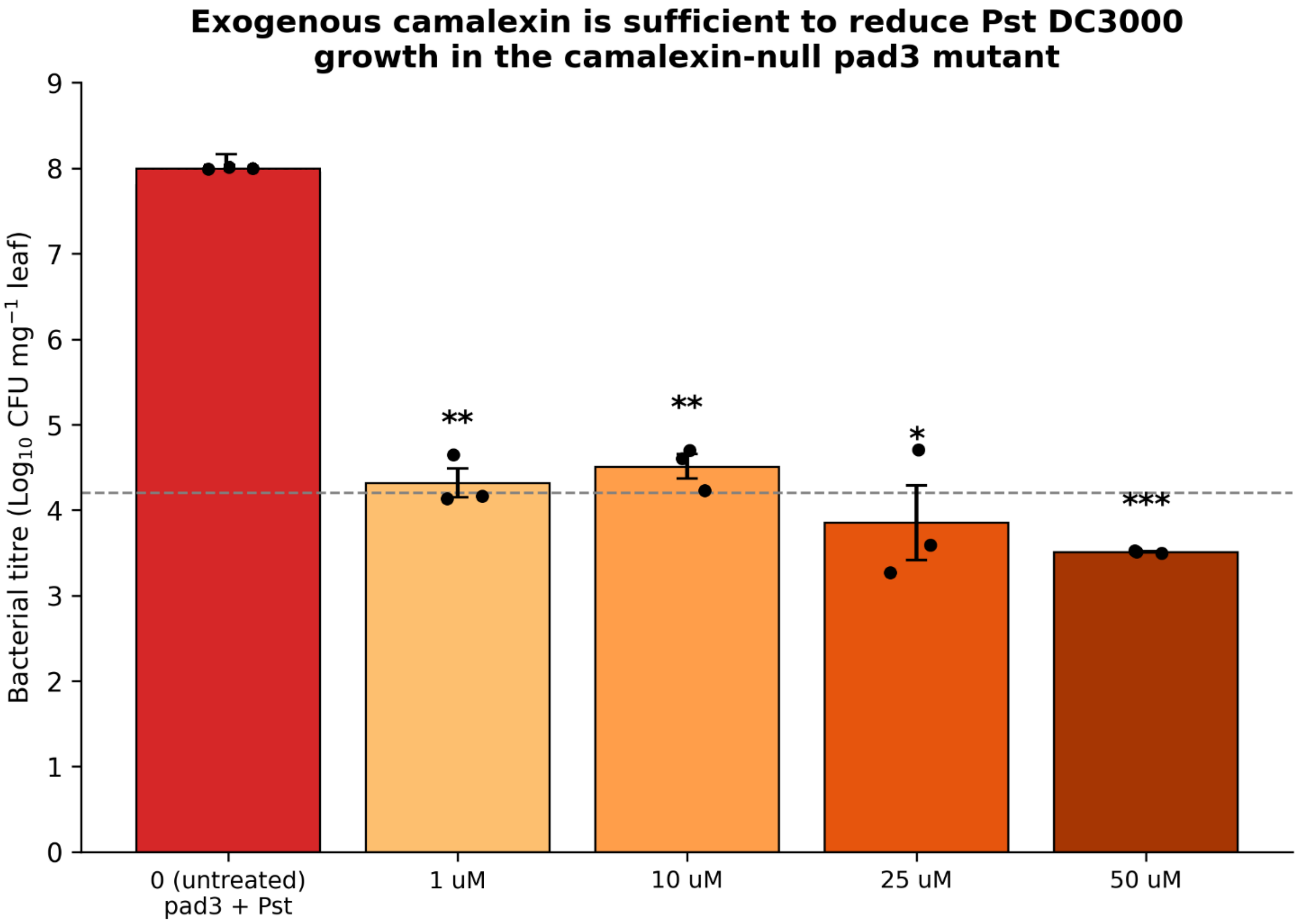

*Significance (Welch t-test) vs untreated pad3 + Pst reference: *** p<0.001, ** p<0.01, * p<0.05.*
Camalexin (1-50 uM) infiltrated 24 h before Pst DC3000 challenge (OD600 = 0.08).
n = 3 leaves/dose, one experiment. Values from bench colony counts
