# Supplementary material for: Airborne Immunity: A Bacterial Volatile Primes Plant Defenses by Unlocking a Phytoalexin Biosynthetic Checkpoint
Source: Plant Cell Environ. 2026 Aug 10;49(11):9070–84. doi: 10.1111/pce.70816 (PMC13646668; doi:10.1111/pce.70816)
Supplement: Supplementary file 2 [file PCE-49-9070-s001.docx]

**Supplementary Table S2. Genes clustered by STRING analysis.**

| **Node degree** | **Gene code** | **Gene name** | **Log2FC** | **Cellular Component** | **Biological process** | **GO term** | **Description** |
| --- | --- | --- | --- | --- | --- | --- | --- |
| 8 | *AT5G10760* | *AED1* | **1.76** | extracellular | Response to biotic stimulus | systemic acquired resistance | APOPLASTIC, EDS1-DEPENDENT 1 Aspartyl protease involved in a homeostatic feedback mechanism regulating systemic immunity. Has only mild or no influence on local defenses. Acts downstream of salicylic acid to suppress systemic immunity. |
| 7 | *AT1G13470* |  | **1.80** | extracellular | Signal transduction | cellular response to organic substance | hypothetical protein (DUF1262) |
| 6 | *AT2G18660* | *PNP-A* | **1.09** | extracellular, cell wall | Response to biotic stimulus | systemic acquired resistance | plant natriuretic peptide A Encodes PNP-A (Plant Natriuretic Peptide A). PNPs are a class of systemically mobile molecules distantly related to expansins; their biological role has remained elusive. PNP-A contains a signal peptide domain and is secreted into the extracellular space. Co-expression analyses using microarray data suggest that PNP-A may function as a component of plant defence response and SAR in particular, and could be classified as a newly identified PR protein. It is stress responsive and can enhance its own expression. |
| 5 | *AT4G03450* |  | **1.04** | cytoplasm | Regulation of defense response | response to molecule of bacterial origin | ankyrin repeat-containing protein mRNA, complete cds [NM_116583] Ankyrin (ANK) repeat-containing proteins occur widely in prokaryotes, eukaryotes, and even in some viruses and play a critical role in plant growth and development, as well as the response to biotic and abiotic stress. |
| 5 | *AT2G32680* | *RLP23* | **1.29** | extracellular, endoplasmic reticulum | Response to biotic stimulus | pathogen-associated molecular pattern receptor signaling pathway, response to molecule of bacterial origin | receptor like protein 23 mRNA, complete cds [NM_128827] Involved in the perception of necrosis and ethylene-inducing peptide 1-like proteins (NLPs), that act as extracellular signals mediating immune activation. Component of the RLP23-SOBIR1-BAK1 complex that mediates NLP-triggered immunity. |
| 4 | *AT4G23140* | *CRK6* | **1.28** | chloroplast, cytosol, plasma membrane | Response to biotic stimulus | response to reactive oxygen species, defense response to bacterium, plant-type hypersensitive response, signal transduction | cysteine-rich receptor-like protein kinase 6 Arabidopsis thaliana receptor-like protein kinase. Naming convention from Chen et al 2003 (PLASMA MEMBRANEID 14756307) |
| 4 | *AT2G14610* | *PR1* | **-1.73** | extracellular region, plant-type cell wall, secretory vesicle | Response to biotic stimulus | defense response, priming of cellular response to stress, response to vitamin B1, response to water deprivation, systemic acquired resistance | pathogenesis-related protein 1, PR1 gene expression is induced in response to a variety of pathogens. It is a useful molecular marker for the SAR response. Though the Genbank record for the cDNA associated to this gene is called 'PR-1-like', the sequence actually corresponds to PR1. Expression of this gene is salicylic-acid responsive. |
| 4 | *AT2G30770* | *CYP71A13* | **-1.58** | endoplasmic reticulum lumen, mitochondrion | Defense response | camalexin biosynthetic process, defense response to bacterium, defense response to fungus, induced systemic resistance, response to bacterium | cytochrome P450, family 71, subfamily A, polypeptide 13 mRNA, complete cds [NM_128630] Indoleacetaldoxime dehydratase; Involved in the biosynthesis of the indole-derived phytoalexin camalexin. Catalyzes the conversion of indole-3- acetaldoxime to indole-3-acetonitrile. Required for resistance to A.brassicicola and B.cinerea; Belongs to the cytochrome P450 family. |
| 4 | *AT1G14880* | *PCR1* | **1.06** | cytoplasm, extracellular region, plant-type vacuole | Response to biotic stimulus | response to salicylic acid, defense response to bacterium | cadmium resistance protein 1 mRNA, complete cds [NM_101357] |
| 4 | *AT1G32350* | *AOX1D* | **-1.76** | mitochondrion | Alternative oxidase activity | alternative oxidase activity, superoxide-generating NADPH oxidase activity | alternative oxidase 1D mRNA, complete cds [NM_102968] |
| 3 | *AT1G01680* | *PUB54* | **2.41** | nucleus, cytoplasm | Ubiquitin-protein transferase activity (GNAT) domain | cellular response to oxygen-containing compound, cellular response to organic substance, signal transduction, import across plasma membrane | Plant U-box type E3 ubiquitin ligase (PUB). |
| 3 | *AT5G52760* | *F6N7.24* | **1.38** | nucleus | Response to biotic stimulus | response to molecule of bacterial origin, response to salicylic acid, small molecule metabolic process | copper transport family protein mRNA, complete cds [NM_124654] |
| 3 | *AT1G15520* | *PDR12* | **-1.86** | plasma membrane | Response to biotic stimulus | response to salicylic acid, abscisic acid transport, response to jasmonic acid, abscisic acid-activated signaling pathway, camalexin | ABCG40 - pleiotropic drug resistance 12 - ABC transporter family involved in ABA transport and resistance to lead. Localizes to plasma membrane. Upregulated by lead. Expressed in leaves, flowers, stomata and roots. |
| 3 | *AT4G37990* | *ATCAD8* | **-1.62** | cytoplasm, plasma membrane | Response to biotic stimulus | plant-type hypersensitive response, cinnamyl-alcohol dehydrogenase activity, lignin biosynthetic process | ARABIDOPSIS THALIANA CINNAMYL-ALCOHOL DEHYDROGENASE 8, ELI3-2, Encodes an aromatic alcohol:NADP+ oxidoreductase whose mRNA levels are increased in response to treatment with a variety of phytopathogenic bacteria. Though similar to mannitol dehydrogenases, this enzyme does not have mannitol dehydrogenase activity. |
| 2 | *AT1G07430* | *AKT1* | **-1.19** | nucleus, chloroplast | Response to endogenous stimulus | negative regulation of abscisic acid-activated signaling pathway, positive regulation of gibberellic acid mediated signaling pathway, protein serine/threonine phosphatase activity | protein phosphatase 2C 3 mRNA, complete cds [NM_100617] INTERACTING PROTEIN PHOSPHATASE 1, HAI2, HIGHLY ABA-INDUCED PP2C GENE 2 |
| 2 | *AT4G04510* | *CRK38* | **1.48** | chloroplast, plasma membrane | Other metabolic processes | protein serine/threonine kinase activity | cysteine-rich receptor-like protein kinase 38 Encodes a cysteine-rich receptor-like protein kinase. |
| 2 | *AT1G71000* | *F15H11.19* | **-1.01** | nucleus, cytoplasm | Response to biotic stimulus | defense response to fungus | chaperone DnaJ-domain containing protein mRNA, complete cds [NM_105769] |
| 2 | *AT4G23310* | *CRK23* | **1.34** | extracellular, plasma membrane | Response to biotic stimulus | defense response to bacterium | Encodes a cysteine-rich receptor-like protein kinase. |
| 2 | *AT1G09080* | *BIP3* | **1.00** | nucleus, chloroplast, cytoplasm, endoplasmic reticulum | Response to stress | endoplasmic reticulum unfolded protein response, chaperone cofactor-dependent protein refolding, ubiquitin-dependent ERAD pathway, response to singlet oxygen | protein BIP3 mRNA, complete cds [NM_100779] BINDING PROTEIN 3, BIP3, HEAT SHOCK PROTEIN 70-13, HSP70-13 Member of the HSP70 family of heat shock proteins. Induced by stress and unfolded protein response. A target of several bZIP proteins. |
| 1 | *AT1G26390* | *ATBBE4* | **-1.49** | cytoplasm, cell wall | Unknown | FAD binding | FAD-binding Berberine family protein;(source:Araport11) |
| 1 | *AT4G33720* | *CAPE3* | **1.82** | extracellular | Response to biotic stimulus | defense response | CAP (Cysteine-rich secretory proteins, Antigen 5, and Pathogenesis-related 1 protein) superfamily protein |
| 1 | *AT5G26170* | *WRKY50* | **1.17** | nucleus | Response to biotic stimulus | jasmonic acid mediated signaling pathway, | putative WRKY transcription factor 50 , member of WRKY Transcription Factor; Group II-c. Involved in jasmonic acid inducible defense responses |
| 1 | *AT3G23120* | *RLP38* | **1.27** | plasma membrane | Unknown | signal transduction | receptor like protein 38 mRNA, complete cds [NM_113213] |
| 1 | *AT1G11040* |  | **-1.72** | chloroplast, cytosol | Unknown | chaperone cofactor-dependent protein refolding | HSP40/DnaJ peptide-binding protein mRNA, complete cds [NM_100977] |
| 1 | *AT1G57780* | *ATHMP11* | **-1.19** | nucleus | Unknown |  | HEAVY METAL ASSOCIATED PROTEIN 11, heavy-metal-associated domain-containing protein mRNA, complete cds [NM_104573] |
| 1 | *AT2G29470* | *GSTU3* | **-4.56** | cytoplasm | Response to chemical | toxin catabolic process, | glutathione S-transferase tau 3 Encodes glutathione transferase belonging to the tau class of GSTs. Naming convention according to Wagner et al. (2002). |
| 1 | *AT3G48640* |  | **1.19** | unknown | Response to biotic stimulus | systemic acquired resistance, cellular response to oxygen-containing compound, signal transduction, response to salicylic acid, response to molecule of bacterial origin | transmembrane protein |
| 1 | *AT4G24410* |  | **-1.40** | unknown | Response to stress | response to oxidative stress | uncharacterized protein mRNA, complete cds [NM_118574] |
| 1 | *AT5G35737* |  | **1.04** | unknown | Unknown | unknown | Ta11-like non-LTR retrotransposon;(source:Araport11). Maternally expressed, imprinted gene. |
| 1 | *AT5G45890* | *SAG12* | **-3.47** | chloroplast, vacuole | Response to biotic stimulus | senescence-associated vacuole, response to auxin, response to sugar, response to ethylene, cysteine-type endopeptidase activity | SENESCENCE-ASSOCIATED GENE 12, Senescence-associated gene 12 (SAG12) encoding a cysteine protease influenced by cytokinin, auxin, and sugars.Localized to special vacuole found during senescence called senescence associated vacuoles which are different from central vacuole in the tonoplast composition and pH. |
