# Supplementary material for: Airborne Immunity: A Bacterial Volatile Primes Plant Defenses by Unlocking a Phytoalexin Biosynthetic Checkpoint
Source: Plant Cell Environ. 2026 Aug 10;49(11):9070–84. doi: 10.1111/pce.70816 (PMC13646668; doi:10.1111/pce.70816)
Supplement: Supplementary file 3 [file PCE-49-9070-s002.docx]

Supplementary Table S1: Primers for genes analysed in this work

| **Name** | **Locus** | **Forward Primer** | **Reverse Primer** |
| --- | --- | --- | --- |
| *ACT2* | AT3G18780 | TGTGCCAATCTACGAGGGTT | TTTCCCGTTCTGCTGTTGTG |
| *AFB2* | AT3G26810 | CGTTGCCAAGAACTGTCCAA | GGCTTTGACGATTGCTCCAA |
| *AXR4* | AT1G54990 | TTGTCACACATTCAGGCAGC | GTTCTTCAGCAACCCGTCTG |
| *BAK1* | AT4G33430 | TGGGAAGAGTGGCAAAAGGA | CAATGATCCAGCCAGACACG |
| *BIK1* | AT2G39660 | GGGGCTTGCTTTTCTTCACA | CCATTGGACCGTCTCTAGCT |
| *BRL1* | AT2G01950 | CTGTTCTTGGTCCGTGAAGC | TCGTACCGCTCTTGGGATAC |
| *BRL2* | AT3G13380 | CTCGGAACAGTCTCATCGGA | AAAGCTCCGGTGGAATCTCA |
| *CaLB-1* | AT1G04540 | AGATCGCACGAAACGGAAAG | CTCTGTTCGCCATCTCTCGA |
| *CaLB-2* | AT2G13350 | CTGTCGTTTTCCTTGGCCTC | ATGGTTGTGTATGAGCCCCA |
| *CML43* | AT5G44460 | GACTTTGCTGCACTCCACAA | CTGATTCCGGAGACCCATCA |
| *CPK28* | AT5G66210 | CTTCAGCCGTAAACCTTGGG | ATCAGTAGCATTCCCGCCTT |
| *CRK6* | AT4G23140 | TCCCAAGGTAAGAGAAGCCG | CGTTTGTAATGGCCGTCGAT |
| *CTR1* | AT5G03730 | ACCTATGAATGCCCCACCAA | AAGATCACACCACGGGATGT |
| *CYP71A13* | AT2G30770 | ATCTTGCTTTGGGTCTGGCA | TGATCCCCATTTGGTCCAGC |
| *CYP71B15 (PAD3)* | AT3G26830 | CAACTCCACTCTTGCTCCCA | TTCGCAGGAACATCGTAGCC |
| *CYP79B2* | AT4G39950 | GAAAAGAGGTTGTGCGGCTC | TTGAAGAAGTCTCGCGAGCA |
| *CYP79B3* | AT2G22330 | ACGACCATGATGCTAGCGAG | ACCAACACCAAAGGCTTCGA |
| *EDS1* | AT3G48090 | CGGATCATGCTTTTGGGCTG | TACCTCCCCGTCTGTGATCC |
| *EF-hand calcium-binding protein family (EFhcbp)* | AT3G03410 | GTTTAGCTCGCCGTTACCG | GAGTTTCGTGAAGTGGCTCT |
| *eEF1Bα2* | AT5G19510 | GTTCACTCCCCAGGGTGCTC | GGGAGCATCATGTTGGCGAG |
| *EIN2* | AT5G03280 | CCGTATGCAGATGTTGGGTG | TCCGTGAACTGTAGCTGGTT |
| *ETR1* | AT1G66340 | GCCATTCCCCGACATTCAAA | CTCGTTTGAACTCACCGTGG |
| *FLS2* | AT5G46330 | TACTCCTTGACAGTGACCGC | TGATGTTGAAGCTGTGGTGC |
| *KAI2* | AT4G37470 | TCTTGGCTGTGAATCCGTCG | TGTGACGGAGGATGACAGGA |
| *MAPKKK14* | AT2G30040 | CGGCGGTTGAGTTTGAGAAA | CTCCGGTCCTTGGTACTCTC |
| *MAPKKK21* | AT4G36950 | AGAGATGGACGGCTGAGATG | TTCGTCTTCTTCGCCACTCT |
| *MAX1* | AT2G26170 | GTATGCAATGTTCCCGGTCC | CGGAGAAGACATCAGGACCA |
| *MAX2* | AT2G42620 | AGTTAGATTGCGGGGACACA | TGGCCAATAATCAAGCTCGC |
| *MPK3* | AT3G45640 | TTGCACGACCCGAATGATGA | TCCAGAGGCTGTTGTTCGAA |
| *MPK6* | AT2G43790 | TCCCTGGACGAGATCATGTC | CAGTGATGGATTGGCGAGGA |
| *PR1* | AT2G14610 | ATGTGGGTTAGCGAGAAGGC | GGCACATCCGAGTCTCACTG |
| *PUB26* | AT1G49780 | TCCATGAGTCGATGCTGGTT | TGCCCAATCTCACTTGACCT |
| *RLP23* | AT2G32680 | TTCACTGGTCCCATTCCTCC | TGTCCGTAAAGAGGCACCAT |
| *SABP3* | AT3G01500 | GCCATTGAATACGCGGTCTT | CCTTTGATCCCACCACATGC |
| *SAUR25* | AT4G13790 | CCGCCCGTTGGATGATTAAA | AGGGTTCTTTGCTGTGTATGT |
| *SAUR61* | AT1G29420 | GCAAAGCGGATTCTATCGGA | ACTACTAGCAGCTCAACCGC |
| *SMAX1* | AT5G57710 | TTTGGGAAACGACGAGCAAG | GTATCAGCGGCTTGGTTCAG |
| *TAA1* | AT1G70560 | GGACGAAGGAAGAGACGGAT | TGTCAGATCCACAACGCTCT |
| *TAR2* | AT4G24670 | GCTCTCTCCCCACATGATGA | GCATCTCCACCCCATCGATA |
| *TPL* | AT1G15750 | CGTGTTGGTTTCGTCTGGAG | GCACTGTTTGGTCTTCCCTG |
| *TSB1* | AT5G54810 | TACTGTGTGTGCCCGTTTTG | GCATCCTTCAATGTCGCTGT |
| *TSB3* | AT5G38530 | AAACACAGCGGTTCCACAAG | GAACTTCCCCATTGACCAGC |
| *UBP6* | AT1G51710 | GAAAGTGGATTACCCGCTG | CTCTAAGTTTCTGGCGAGGAG |

| **Name** | **Locus** | **Forward Primer** | **Reverse Primer** |
| --- | --- | --- | --- |
| *WRKY22* | AT4G01250 | TAGCGGTGGGAGATCTTGAC | CGGCTGCGAATTCCTCTAAC |
| *WRKY29* | AT4G23550 | AGAAATGAGCGTTGCGGAAA | AGTTCATCGAAGTCACCGGT |
| *WRKY33* | AT2G38470 | CGAAGGGAAACACAACCACG | CGAATCCTGTGGTGCTCTGT |
| *WRKY41* | AT4G11070 | TCCAAAGATCGGACGGTGAT | AGACTGGCCCTGAGATTCTG |
| *XLG2* | AT4G34390 | GCAATGGTTCTTGAGGCACA | TCCCACATTGCCTGAAGACT |
| *YUC1* | AT4G32540 | GCAGATCCATACTTCCCCGA | TTCGCCGCTTTTGTACTCAC |
| *YUC3* | AT1G04610 | GGCGAGGATTATCTTGGGGA | GGTAACGCTCCGATGTCAAG |
| *YUC9* | AT1G04180 | AAGACGCCGGTTCTTGACAT | GACCATCTACGAGCTCCACG |
